# Minimally Invasive Management of Subclavian Artery Catheter Misplacement: The New Standard?

**DOI:** 10.3390/jcm14082650

**Published:** 2025-04-12

**Authors:** Lukas Lenhart, Alexander Loizides, Malik Galijasevic, Maximilian Lutz, Martin Freund, Elke R. Gizewski, Astrid E. Grams

**Affiliations:** Department of Radiology, Medical University of Innsbruck, 6020 Innsbruck, Austria; lukas.lenhart@i-med.ac.at (L.L.); malik.galijasevic@i-med.ac.at (M.G.); maximilian.lutz@i-med.ac.at (M.L.);

**Keywords:** subclavian artery, catheter misplacement, percutaneous closure device, Angio-Seal, standardized algorithm

## Abstract

**Background**: The accidental puncture of the supra-aortal arteries during central venous catheterization is a rare but potentially life-threatening complication. Traditional management often requires open surgical repair, which is associated with significant morbidity. This study evaluates an endovascular approach for managing such cases using an Angio-Seal™ vascular closure device (Terumo Medical Corporation, Somerset, NJ, USA). **Methods**: Between January 2010 and December 2024, 47 patients with misplaced catheters in supra-aortal arteries were treated at our institution. Of these, 37 cases involving subclavian artery catheter misplacements were managed using a standardized algorithm and form the focus of this study. Additional interventions, such as stent graft placement or balloon inflation, were performed as needed. **Results**: Primary technical success was achieved in 86.5% of cases. Four patients required stentgrafts and one balloon inflation for persistent extravasations. One patient developed a small subclavian pseudoaneurysm, which resolved spontaneously. Primary assisted technical success and clinical success rates were both 100%. **Conclusions**: This study demonstrates the efficacy and safety of our minimally invasive endovascular approach for managing subclavian artery catheter misplacements. With a high success rate, low complication rate, and the avoidance of open surgery, this algorithm offers a promising alternative for treating this rare but serious complication of central venous catheterization.

## 1. Introduction

The accidental puncture of the supra-aortal arteries is a rare but potentially life-threatening complication of central venous catheterization. Catheter misplacements are typically detected immediately through arterial blood aspiration, but in some cases, they may only be discovered during post-procedural radiographic evaluation [1]. While the incidence of inadvertent arterial puncture during subclavian vein catheterization is reported to be between 3.1 and 4.9%, the consequences can be severe, including hemorrhage, hemothorax, pseudoaneurysm formation, and stroke [2,3]. The traditional management of such injuries often requires open surgical repair, which can be challenging due to the anatomical location and associated morbidity.

For the treatment of misplaced arterial catheters, endovascular techniques have emerged as promising alternatives for managing subclavian artery injuries [4,5,6,7,8]. These minimally invasive approaches, including the use of balloon tamponades, covered stents, vascular closure devices, and embolization techniques, offer several advantages over open surgery, including reduced surgical trauma, lower blood loss, and shorter hospital stays [9].

The growing body of literature on this topic demonstrates the efficacy and safety of endovascular treatments for subclavian artery injuries. Studies have reported high technical success rates, with initial endovascular stent placement being successful in up to 96.9% of cases [10]. Moreover, follow-up data suggest good long-term patency rates and a low incidence of procedure-related complications [9,10]. The off-label application of Angio-Seal™, a collagen-based vascular closure device (Terumo Medical Corporation, Somerset, NJ, USA), for managing inadvertent subclavian artery punctures is emerging as a minimally invasive and effective approach showing promising results [4,11,12,13,14,15,16]. Angio-Seal™ offer rapid hemostasis and thus may be preferred for uncomplicated cases, whereas stent placement might be preferred for complex scenarios involving larger injuries, pseudoaneurysms, or stenosis by providing structural support and maintaining patency. Recent case reports and small case series highlight the successful use of Angio-Seal^TM^ in managing iatrogenic subclavian artery punctures achieving complete hemostasis [17,18]. Successful outcomes in supra-aortic arterial injuries were noted even in use with larger sheath sizes supporting the use of closure devices [19]. Confirming these findings, the application of vascular closure devices like Perclose ProGlide (Abbott Vascular Inc., Santa Clara, CA, USA) [20,21] achieved clinical success in patients with subclavian artery injury. Dornbos et al.’s systematic review in 2019 [22] indicated that vascular closure devices are frequently used as the primary method for repairing subclavian and brachiocephalic arterial injuries, demonstrating high effectiveness (94%) and few complications. These findings suggest that vascular closure devices offer a valuable alternative to more invasive open surgical repair, providing a quick and safe means to control bleeding in inadvertent supra-aortic arterial injuries following iatrogenic subclavian cauterization.

The objectives of this study are to present and evaluate a standardized endovascular algorithm for managing misplaced subclavian artery catheters using the Angio-Seal™ vascular closure device. This algorithm, developed from the existing literature and clinical experience, aims to provide a structured approach for safe and effective intervention. This study seeks to assess the safety and efficacy of this algorithm by evaluating technical success rates, complication rates, and procedural outcomes in a series of patients. By building on evidence supporting endovascular techniques and the off-label use of Angio-Seal™, this study aims to contribute to a more standardized and validated method for addressing this challenging clinical scenario.

## 2. Materials and Methods

### 2.1. Study Participants

Between January 2010 and December 2024, 47 patients were referred to our interventional radiology department for the removal of misplaced central vein catheters in supra-aortal arteries. From this cohort, 10 cases required individualized approaches due to atypical presentations. Two patients with accidental vertebral artery catheterization were treated using coils and stentgrafts. One patient with a subclavian artery pseudoaneurysm (post-catheter removal) was treated with a stentgraft. A stentgraft was primarily used for a misplaced catheter in the right subclavian artery in one patient. An 8F Star-Close device was employed for a right subclavian artery catheter in one case. A stentgraft addressed a prior brachiocephalic trunk puncture in one patient. Four patients underwent open surgery: two for common carotid artery involvement, one for brachiocephalic trunk, and one for proximal right subclavian artery. The remaining 37 patients, all with subclavian artery catheter misplacements, form the core focus of this work. For all patients with subclavian artery catheter misplacements, the misplaced catheters were still in situ at the time of intervention. These cases were managed using our standardized step-up approach.

### 2.2. Management of Accidental Subclavian Artery Catheterization

All procedures were conducted within an angiographic suite. The choice of local or general anesthesia depended on the severity of the patient’s underlying medical conditions. Throughout the interventions, patient monitoring included electrocardiography, blood pressure measurements, and pulse oximetry. A right femoral approach was consistently used to gain transarterial access. Diagnostic angiography was performed to precisely locate the catheter within the artery, as well as the position of its tip. Our standardized approach for managing misplaced catheters in the subclavian artery is based on clinical experience and the existing literature [4]. The procedure involves the following steps:

1. Establish inguinal arterial access via an ultrasound-guided puncture of the common femoral artery.
2. Advance a catheter in the affected subclavian artery and perform angiographic contrast series in at least two projections to visualize the exact position of the misplaced catheter or the presence of any complications such as dissection or hemorrhage.
3. Measure the vessel diameter and prepare an appropriate angioplasty balloon in the angio-suite as a precautionary measure.
4. Maintain the guide wire in the affected subclavian artery throughout the procedure.
5. Insert an Amplatz wire into the misplaced catheter, and then remove the catheter.
6. Depending on the catheter’s diameter, deploy a 6F or 8F Angio-Seal™ vascular closure device according to the manufacturer’s instructions.
7. Conduct a follow-up angiographic series to confirm successful closure.
8. If hemorrhage or pseudoaneurysm is detected, inflate the prepared angioplasty balloon for at least three minutes. For persistent hemorrhage, deploy an appropriately sized stentgraft, taking care to preserve the vertebral artery origin.
9. Remove the inguinal access if no complications are observed.

### 2.3. Angio-Seal Device

The Angio-Seal™ VIP Vascular Closure Device (Terumo Medical Corporation, Somerset, NJ, USA) is a collagen-based, plug-mediated system designed for arterial closure. It comprises three biodegradable components: an anchor, a small bovine collagen plug, and a traction suture for secure fixation. While the device is designed to be introduced over a 0.035-inch guide wire, we often found the included wire inadequate due to its short length and flexibility. Consequently, we opt for a 0.035-inch stiff Amplatz guide wire. All components of the Angio-Seal device are fully bioabsorbable, dissolving within 60–90 days post-procedure [4,23,24]. Studies have shown that the Angio-Seal^TM^ vascular closure device is effective in sealing large-caliber arteriotomies with low complication rates [25].

### 2.4. Balloon and Stentgraft Applications

Endovascular repair was performed using Angio-Seal™ devices, temporary occlusion with balloon catheters, or balloon catheters followed by stentgraft placement. Stentgrafts were reserved for cases where balloon occlusion or Angio-Seal™ device repair proved unsuccessful. Completion angiography was routinely performed in all patients to confirm arterial patency, normal vessel appearance, and the absence of extravasation. During balloon catheter applications, the catheters were positioned across the catheter entry site, followed by simultaneous balloon inflation and catheter removal. Following treatment, the balloon catheter was replaced with a diagnostic catheter for completion angiography. For stentgraft applications, a 0.035-inch stiff Amplatz guide wire and 10–12 French sheath were utilized to gain access through the right femoral artery and facilitate device introduction. Stentgrafts were deployed under fluoroscopic control, using roadmap techniques and anatomical landmarks. Completion angiography was performed post-stentgraft placement to document the correct stentgraft position, the status of branch arteries, and the presence of any endoleaks. Femoral puncture sites were closed by endovascular repair. Patients receiving a stentgraft were prescribed lifelong antiplatelet therapy with a daily dose of 100 mg acetylsalicylic acid. Stentgraft patency was monitored through ultrasound studies or clinical examination.

### 2.5. Follow-Up Protocol and Definitions

The follow-up protocol included comprehensive pre-discharge assessments comprising clinical examination, laboratory tests, and chest radiography, with CT performed as needed. While there was no defined follow-up protocol after discharge, patients were monitored for a period of three months. Given the patients’ severe comorbidities, they underwent close observation with frequent imaging for their underlying conditions.

Study definitions distinguished between primary technical success (successful misplaced catheter removal in first attempt without secondary intervention), primary assisted technical success (successful secondary intervention during the same session), and clinical success (catheter removal without procedure-related morbidity, mortality, or surgical reconstruction). These definitions were evaluated on an intent-to-treat basis, ensuring the comprehensive and standardized assessment of procedural outcomes [4].

## 3. Results

In this study of 47 individuals, the mean age was 61.2 years (range 20–84 years), with 55.3% being women. Subclavian mispunctures accounted for 78.7% of cases, with 45.9% on the left side and 54.1% on the right side. The puncture location was lateral to the thyrocervical trunk in 73% of cases, between the thyrocervical trunk and vertebral artery in 21.6%, and medial to the vertebral artery in 5.4%. Misplaced catheter sizes ranged from 8.5 to 12 French. A total of 19 patients (51%) had 8.5-French misplaced catheters, while 7 patients (19%) had 12-French misplaced catheters in the subclavian artery. For the remaining 11 patients (30%), the catheter size information was not available. The misplaced catheters were fairly evenly distributed between the left and right subclavian arteries, with 46% in the left subclavian artery and 54% in the right subclavian artery. An 8-French Angio-Seal™ was used in 33 patients (89%), while a 6-French Angio-Seal™ was used in the remaining 4 patients (11%). Femoral closure devices were used in 30 patients (81% of cases). Table 1 provides detailed information about the accidental puncture sites, subsequent interventions, and endovascular success for the 37 patients with subclavian mispunctures.

### 3.1. Technical Success Rates

**Figure 1:**
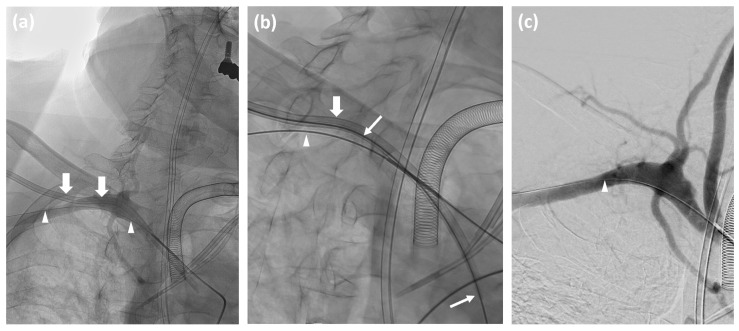
(**a**) Non-subtracted angiography of a 66-year-old male shows a misplaced 12F 3-lumen central venous catheter (CVC) in the right peripheral subclavian artery (thick arrows), extending to the ascending aorta; correctly positioned gastrointestinal and tracheostomy tubes, and correctly placed left-sided CVCs; 0.035-inch “lock wire” (arrowheads) through a 5F MAP1 catheter in the brachiocephalic trunk. (**b**) Stiff Amplatz guide wire (thin arrows) inserted via a misplaced CVC in the right subclavian artery, used for CVC removal. (**c**) Post-procedure DSA after CVC removal and Angio-Seal™ deployment shows complete closure without complications.

The primary technical success rate, defined as the successful removal of the misplaced catheter on the first attempt without requiring secondary intervention, was 86.5% (Figure 1).

**Figure 2:**
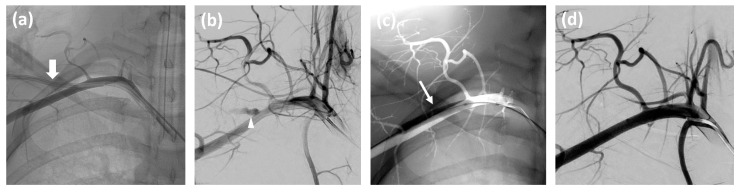
Images from a 20-year-old male patient: (**a**) non-subtracted angiography shows the misplaced catheter in the right subclavian artery (thick arrow). (**b**) Subtracted selective angiography after catheter removal shows contrast extravasation (arrowhead). (**c**) Balloon inflation (thin arrow) at the arterial puncture site. (**d**) Subtracted angiography confirms the successful and complete closure of the puncture site.

Post-procedural hemorrhage occurred in 13.5% of cases (five patients) following Angio-Seal™ deployment. Persistent extravasation was managed successfully in all instances: balloon inflation resolved bleeding in one case (Figure 2), while stentgraft placement was required in four others. Additionally, one patient developed a small subclavian pseudoaneurysm at the puncture site, which resolved spontaneously without intervention.

Both primary assisted technical success and clinical success rates reached 100%, demonstrating the complete resolution of complications through secondary interventions and the achievement of intended clinical outcomes for all patients in the cohort.

### 3.2. Post-Procedure Morbidity and Mortality

One patient developed an inguinal pseudoaneurysm, which was successfully treated using ultrasound-guided fibrin glue injection without any complications. Within one week following the procedure, five patients in the cohort died. It is crucial to note that these deaths were attributed to pre-existing medical conditions. They were not related to the misplacement or the interventional procedure itself.

## 4. Discussion

In the present study, we provide evidence that Angio-Seal™ is an effective approach for managing misplaced catheters in the subclavian artery, presenting a large case series with 37 patients. The procedure had an 86.5% primary technical success rate, with 13.5% experiencing hemorrhage post-Angio-Seal™ deployment, managed through balloon inflation or stentgraft application. The study achieved 100% primary assisted technical success and clinical success rates.

The 86.5% primary technical success rate with Angio-Seal™ aligns with previous reports using vascular closure devices of iatrogenic arterial catheterizations [6,11,13,16], and our management of complications (13.5% hemorrhage rate) through balloon inflation or stentgraft application is consistent with strategies described by Melas et al. and Shetty et al. [7,15]. Our 100% clinical success rate parallels outcomes reported by Cohen et al. and Szkup [11,16], underscoring the reproducibility of these techniques. Importantly, our findings contribute to the growing body of evidence supporting minimally invasive, endovascular approaches for managing subclavian artery mal catheterization [8,12,14]. While our higher success rate compared to Melas et al. (69.2%) may reflect refined expertise or patient selection, all studies converge on the critical role of endovascular salvage techniques in achieving ultimate success. A similar study by Verloh et al. demonstrated a primary technical success rate of 80% (four out of five patients) [26], suggesting consistency in procedural outcomes across different clinical settings. The primary assisted technical success rate of 100% in this study also mirrors findings from other reports, which demonstrated that stentgrafts effectively resolved complications when initial closure methods failed, achieving a similar success rate [4].

The results of this study underscore the reliability and significant advantages of endovascular techniques as a minimally invasive alternative to surgical approaches for managing accidental subclavian artery catheterizations. Compared to traditional surgical methods, endovascular management offers high technical success rates and low procedural morbidity, and avoids open surgical intervention. This endovascular approach reduces surgical trauma, shortens the length of in-hospital stay (i.e., 9.7 days less), and lowers the risk of complications such as infection and excessive blood loss (i.e., 1368 mL less) [9]. Endovascular techniques allow for precise interventions through smaller access points, minimizing patient discomfort and morbidity. The use of devices like the Angio-Seal™ vascular closure system, combined with adjunctive measures, when necessary, provides a versatile solution tailored to individual patient needs. This approach is particularly beneficial for patients with severe comorbidities who may not tolerate invasive procedures well. The 100% clinical success rate observed aligns with the most optimistic reports in the current medical literature [4,27], reinforcing the growing role of endovascular techniques as the preferred approach for managing such complications.

The Angio-Seal™ device offers distinct advantages over stentgrafts for managing central venous catheter misplacement in the subclavian artery. Its minimally invasive nature, the preservation of native vascular anatomy, faster deployment, and the avoidance of long-term anticoagulation make it an attractive option [28,29]. The application of stentgrafts carries risks of migration, thrombosis, and in-stent restenosis, whereas Angio-Seal™ may eliminate the need for permanent implants, reduces procedural costs, and maintains flexibility for future interventions [26,30,31]. Additionally, its absorbable design minimizes complications such as vessel distortion or compromised blood flow to critical branches [29]. While suitability relies on factors like injury location and device–catheter size compatibility, Angio-Seal™ may provide a safer, quicker, and more cost-effective solution for uncomplicated cases requiring immediate closure [28,29]. Therefore, closure with Angio-Seal™ is the first attempt in our approach.

While endovascular treatment has become a favored approach for managing many vascular injuries due to its minimally invasive nature, certain anatomical sites, such as the carotid artery and the brachiocephalic trunk, present unique challenges that may render endovascular techniques less ideal. These regions are characterized by complex vascular anatomy, including tortuosity, angulation, and proximity to critical structures, which can complicate catheter navigation and device deployment. For instance, the brachiocephalic trunk’s deep location and potential for significant atherosclerosis or stenosis may increase the risk of complications such as dissection or embolization during endovascular procedures. Similarly, interventions in the carotid artery carry a heightened risk of cerebral ischemia due to plaque detachment or inadequate brain protection during stenting or closure device application [32,33].

The observed complications, including hemorrhage and pseudoaneurysm formation, are consistent with known risks associated with vascular closure devices. Extravasation following Angio-Seal^TM^ deployment can arise from device-related or anatomical considerations. Multiple puncture attempts during catheter placement, device malfunction, or incorrect anchor placement, potentially due to calcified plaques, can lead to extravasation [34]. Furthermore, intraluminal collagen plug deployment may occur in smaller vessels, elevating the risk of malfunction. While our study focuses on iatrogenic subclavian artery injuries from catheter misplacement, complications like pseudoaneurysms can also arise from traumatic, degenerative, or spontaneous causes. These conditions share arterial wall disruption as a common mechanism. However, iatrogenic cases present distinct challenges due to anticoagulation use, delayed detection, and the need for minimally invasive approaches in high-risk patients. Persistent hemorrhage requiring stentgraft application has been highlighted in prior studies as a critical secondary intervention when initial closure methods fail [4,35]. Similarly, the resolution of pseudoaneurysms through balloon remodeling or spontaneous healing aligns with reports suggesting that small pseudoaneurysms can often be managed conservatively or with minimally invasive techniques [36]. Importantly, the absence of procedure-related morbidity and mortality in this cohort further supports the safety profile of endovascular management. The report of five patient deaths within one week, all attributed to pre-existing conditions rather than procedural complications, is consistent with outcomes seen in critically ill populations undergoing central venous catheterization. The literature consistently emphasizes that mortality in such cases is typically related to underlying comorbidities rather than the intervention itself [37,38], highlighting the importance of careful patient selection and monitoring in high-risk groups.

Limitations to this study prevent us from drawing definitive conclusions: The retrospective character of this study limits the strength of the evidence and introduces potential bias. Although the sample size is to our knowledge the largest ever reported, it is still relatively small, particularly for subgroup analyses of complications such as hemorrhage or pseudoaneurysms, reducing the statistical power to assess rare events. Additionally, the inclusion of patients with significant comorbidities and the absence of a control group make it difficult to generalize the findings or compare the efficacy of this approach to alternative treatments, such as open surgery or other endovascular techniques. The off-label use of the Angio-Seal™ device for subclavian artery closure introduces variability in outcomes and raises questions about its broader applicability. Furthermore, the lack of long-term follow-up data limits our understanding of the durability of stentgrafts and the resolution of complications over time.

## 5. Conclusions

In conclusion, this study highlights an effective approach for managing misplaced catheters in the subclavian artery, presenting one of the largest case series to date with 37 patients. The technique achieved a high immediate success rate and demonstrated its safety with a low complication rate. The combination of an Angio-Seal™ vascular closure device at the puncture site with transfemoral access and catheter placement in the affected subclavian artery represents a unique dual-access strategy not commonly reported in the previous literature. Furthermore, the versatility of this minimally invasive approach is evident in its adaptability, as additional interventions such as stentgraft insertion or balloon inflation were successfully employed when needed. By offering a less invasive alternative to surgical repair, this method reduces patient trauma and recovery time while maintaining high efficacy and safety. Immediate vascular closure with Angio-Seal™ use alone may represent a quicker and more cost-effective solution for uncomplicated cases compared to stentgrafts. Overall, these findings provide evidence for a potentially new standard approach for managing this rare but serious complication of central venous catheterization. Future research should focus on evaluating long-term outcomes, exploring broader applications to other supra-aortal arteries, and conducting comparative studies with traditional surgical approaches to further validate this promising alternative to open surgery.

## Figures and Tables

**Table 1:** Patient characteristics and procedure results.

Demographics			Procedure Details				Success			Closure Device	Comments
No.	Age	Sex	Side	Localization *	Catheter Size (F)	Angio-Seal (F)	PTS	PATS	CS		
1	29	F	L	3	8.5	8	Y	N	Y	Y	N
2	84	F	R	2	8.5	8	Y	N	Y	Y	N
3	59	F	L	3	8.5	8	Y	N	Y	Y	N
4	84	F	L	3	N/A	6	N	S	Y	Y	extravasation
5	56	F	L	3	8.5	6	Y	N	Y	Y	N
6	76	F	R	2	8.5	8	N	S	Y	Y	extravasation
7	67	M	L	3	8.5	8	Y	N	Y	Y	N
8	42	F	R	2	8.5	6	Y	N	Y	Y	N
9	74	F	R	2	8.5	8	Y	N	Y	Y	N
10	35	F	R	3	8.5	8	Y	N	Y	Y	N
11	76	M	R	3	8.5	8	Y	N	Y	Y	N
12	74	F	L	2	N/A	6	Y	N	Y	Y	N
13	66	M	R	3	12	8	Y	N	Y	Y	N
14	68	M	R	3	8.5	8	Y	N	Y	Y	inguinal PSA
15	68	F	L	3	8.5	8	Y	N	Y	Y	N
16	57	M	L	3	8.5	8	Y	N	Y	Y	N
17	62	M	L	2	12	8	Y	N	Y	Y	N
18	59	F	L	3	12	8	Y	N	Y	Y	N
19	42	M	R	3	12	8	N	S	Y	Y	extravasation
20	57	M	L	3	N/A	8	Y	N	Y	Y	N
21	72	M	R	3	8.5	8	Y	N	Y	N	N
22	70	F	R	2	12	8	Y	N	Y	Y	N
23	47	M	R	3	N/A	8	Y	N	Y	N	subclavian PSA
24	20	M	R	1	N/A	8	N	B	Y	Y	extravasation
25	48	M	R	3	8.5	8	N	S	Y	N	extravasation
26	74	F	L	3	8.5	8	Y	N	Y	N	N
27	70	F	L	3	8.5	8	Y	N	Y	Y	N
28	61	F	L	3	8.5	8	Y	N	Y	N	N
29	58	M	L	3	12	8	Y	N	Y	N	N
30	61	M	R	3	N/A	8	Y	N	Y	Y	N
31	65	F	R	3	N/A	8	Y	N	Y	N	N
32	69	M	R	2	N/A	8	Y	N	Y	Y	N
33	80	F	R	3	8.5	8	Y	N	Y	Y	N
34	56	M	R	3	N/A	8	Y	N	Y	Y	N
35	55	F	R	3	12	8	Y	N	Y	Y	N
36	70	M	L	3	N/A	8	Y	N	Y	Y	N
37	52	F	L	1	N/A	8	Y	N	Y	Y	N

* 1 = proximal to vertebral artery; 2 = between vertebral artery and thyrocervical trunk; 3 = lateral to thyrocervical trunk; CS, clinical success; PATS, primary assisted technical success; PTS, primary technical success; S, stentgraft; B, balloon; F, French; PSA, pseudoaneurysm.

## Data Availability

The data presented in this study are available on request from the corresponding author.
